# Rapid Identification of Soybean Varieties by Terahertz Frequency-Domain Spectroscopy and Grey Wolf Optimizer-Support Vector Machine

**DOI:** 10.3389/fpls.2022.823865

**Published:** 2022-03-11

**Authors:** Xiao Wei, Dandan Kong, Shiping Zhu, Song Li, Shengling Zhou, Weiji Wu

**Affiliations:** ^1^College of Biosystems Engineering and Food Science, Zhejiang University, Hangzhou, China; ^2^College of Engineering and Technology, Southwest University, Chongqing, China; ^3^China Tianjin Grain and Oil Wholesale Trade Market, Tianjin, China

**Keywords:** soybean, DPLS, PSO-SVM, GWO-SVM, THz spectroscopy

## Abstract

Different soybean varieties vary greatly in their nutritional value and composition. Screening for superior varieties is also essential for the development of the soybean seed industry. The objective of the paper was to analyze the feasibility of terahertz (THz) frequency-domain spectroscopy and chemometrics for soybean variety identification. Meanwhile, a grey wolf optimizer-support vector machine (GWO-SVM) soybean variety identification model was proposed. Firstly, the THz frequency-domain spectra of experimental samples (6 varieties, 270 in total) were collected. Principal component analysis (PCA) was used to analyze the THz spectra. After that, 203 samples from the calibration set were used to establish a soybean variety identification model. Finally, 67 samples from the test set were used for prediction validation. The experimental results demonstrated that THz frequency-domain spectroscopy combined with GWO-SVM could quickly and accurately identify soybean varieties. Compared with discriminant partial least squares (DPLS) and particles swarm optimization support vector machine, GWO-SVM combined with the second derivative could establish a better soybean variety identification model. The overall correct identification rate of its prediction set was 97.01%.

## Introduction

Soybean is one of the most important raw materials for oil and feed (Herman et al., 2018; Kumar et al., 2021; Wei et al., 2021a). Differences in soybean varieties lead to significant differences in their protein, fat, and other constituent contents (Wang et al., 2020a,b). At the same time, soybean variety screening has a crucial impact on the quality of soybean products. Currently, common methods for soybean variety identification include simple sequence repeat (SSR) molecular marker assays (Lu et al., 2018; Wen et al., 2020) and detection of soybean components to determine their varieties (Larsen, 1967; Ujiie et al., 2005), among others. Although the accuracy of the above methods is relatively high, and the sensitivity is relatively strong, and the application is relatively wide. However, they have problems such as relatively long time consuming, relatively low efficiency, and relatively complicated detection process. In recent years, Near-infrared spectroscopy (NIRS) technology has been introduced for the detection of agricultural varieties (Lun Liu et al., 2010; Teye et al., 2014). Compared to SSR molecular marker assays and the soybean component-based detection variety method, the NIRS technology has the advantage of not requiring pre-treatment of samples. Nevertheless, it has limitations in detecting soybeans with surface defects (Zhu et al., 2010) and limited detection accuracy (Chen et al., 2019; Rong et al., 2020). Hence, it is essential to study a rapid and accurate identification method suitable for different varieties of soybeans.

Terahertz (THz) spectroscopy has unique advantages in soybean variety identification (Wei et al., 2020, 2021b). THz spectroscopy is based on coherent THz pulses generated by ultrafast optics. It is a broadband linear spectral detection technique. Due to the weak interaction forces between biological macromolecules (hydrogen bonding, van der Waals forces), backbone vibrations and dipole rotations, etc. fall right in the THz spectral range. At the same time, THz pulses have a good temporal resolution (on the order of picoseconds). Therefore, THz spectroscopy technology is currently cross-cutting frontier research that is received great attention (Yang et al., 2016). Currently, there has been some research on the identification of agricultural product and food varieties through THz spectroscopy. For instance, Wu et al. (2020) proposed a method for sesame oil variety identification based on THz time-domain spectroscopy. Eventually, the identification model using radial basis kernel function achieved a 100% identification rate. Yang et al. (2021) used THz spectroscopy and competitive adaptive reweighted sampling (CARS) combined with support vector machine (SVM) for the detection of high oil and common maize. Ultimately, the model identification rate could reach 100%. Ge et al. (2015) applied THz spectra and partial least squares regression (PLSR) models to discriminate wheat varieties. Eventually, the prediction accuracy of the optimized model using interval partial least squares was significantly improved. The related coefficient of prediction set for their wheat variety detection model was 0.992. Li et al. (2019) used THz spectra and a neural network learning vector quantization model for qualitative identification of maize varieties. By changing the ratio of dividing the training and prediction sets, the final prediction set had a 100% discrimination rate. Luo et al. (2019) conducted a study on soybean variety identification using THz spectroscopy and integrated learning methods. The studied pre-processing methods, integrated classifiers, and comparison methods. Finally, the average accuracy of the proposed model was 89.29%. In summary, there are relatively few reports on soybean variety identification based on THz frequency-domain spectroscopy, and such related studies still have some academic value and significance.

The objective of the study was to analyze the feasibility of THz frequency-domain spectroscopy and chemometrics to identify soybean varieties. Also, a soybean variety identification model based on the grey wolf optimizer-support vector machine (GWO-SVM) was proposed. After different pre-processing methods, the discrimination results of three [discriminant partial least squares (DPLS), particles swarm optimization-support vector machine (PSO-SVM), and GWO-SVM] soybean variety identification models were compared. The most appropriate pre-processing method for each variety identification model was selected.

## Materials and Methods

### Experimental Materials

Soybean samples of six varieties (HuaiDou 2, LuDou 1, NiuMoHang, LuDou 4, HeDou 12, QiHang 34, abbreviated as HD_2_, LD_1_, NMH, LD_4_, HD_12_, QH_34_) were collected for this experiment. Among them, HD_2_, LD_1_, and NMH were each two batches. LD_4_, HD_12_, and QH_34_ were each three batches. Each batch of soybean samples was weighed 50 g to perform subsequent experiments. Eighteen experimental samples were prepared for each batch of soybean samples. A total of 270 samples were prepared. The soybean samples used in the experiments were collected by the Quality Inspection Center of the Tianjin Grain and Oil Wholesale Trading Market in China. The quality inspection center conducted the soybean sample collection in strict accordance with soybean varieties. This made the subsequent soybean variety identification experiments in this paper more rigorous and accurate.

### Terahertz Spectroscopy Experimental Equipment

In the experiment, the THz spectroscopy equipment from EKSPLA was used for the spectral data acquisition of the experimental sample. The equipment used the FF50 femtosecond laser as the ultrashort pulse laser source. The central wavelength was 1,064 nm, and the pulse duration was less than 150 fs. The repetition frequency was about 80 MHz, and the output power was greater than 40 mW, and the spot diameter was less than 2 mm. The equipment used low-temperature-grown gallium arsenide as the generator and detector of the THz wave. The optical distance between the generator and detector was about 62.5 cm. The pump light source was divided into two beams of 55:45 by the beam splitter after passing through the half-wave plate. The first pump light was guided by the reflector through the fast delay line. After that, it was then directed through a set of the optical lens into the THz emitter to excite the THz pulse. The second part of the pump laser beam passed through the slow delay line. Afterward, it was guided to the THz detector by a reflector. The THz pulse was incident vertically on the experimental sample through a metal parabolic mirror. Then, it was focused to reach the THz detector, where it converged with the second part of the beam. The beam signal was fed to the lock-in amplifier for amplification. Finally, The THz time-domain spectra of the experimental samples were obtained.

### Experimental Sample Preparation and Terahertz Spectrum Acquisition

Firstly, the soybean samples were dried in a 40°C drying oven for 3 h. This reduced the moisture in the samples during transportation and storage, thus reducing the effect of moisture in the soybean samples on the experiment. Afterward, the soybean samples were crushed using a pulverizer. The crushed samples were then further ground through a mortar and pestle to obtain the soybean sample powder. Secondly, the soybean sample powder was filtered through the sieve with pore sizes of 0.074 mm. Later, the filtered sample powder was taken and added to polyethylene powder (sample powder and polyethylene powder were mixed in the ratio of 7:3). The two powders were mixed thoroughly to obtain the experimental sample powder. Finally, the experimental sample powder was weighed 135 mg using a precision balance. The sample powder was pressed under the pressure of 20 MPa to form a flake with a thickness of about 1 mm. The surface of the flakes was ensured to be smooth. The room temperature of the THz spectroscopy acquisition laboratory was controlled at 25°C. Nitrogen gas was charged into the THz spectroscopy experimental equipment before the start of the experiment. The relative humidity in the experimental equipment was kept below 5% at all times. During the experiment, the experimental samples were loaded into the sample holder and their THz time-domain spectra were scanned. Each experimental sample was scanned 256 times, and a total of 6 sample points were scanned. The THz spectra of the six sample points were averaged. The THz time-domain spectra were converted to THz frequency-domain spectra by the device software. The THz spectra were acquired by the software that came with the THz spectroscopy experimental equipment.

**FIGURE 1:**
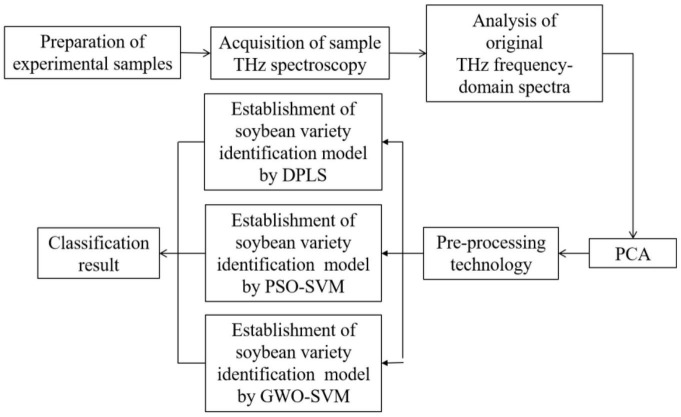
Research flowchart.

The main research flow chart of this paper is shown in Figure 1.

### Theory

#### Principal Component Analysis

Principal Component Analysis (PCA) is a common way of data analysis. In order to extract the main features and information of the THz spectra of experimental samples, PCA is often performed on the spectral data (Wang et al., 2022). The main purpose of PCA is to reduce the dimensionality of THz spectral data as a way to exclude the numerous chemical information that overlaps each other. It mainly highlights the similarities and differences of the data. This is because data identification is difficult to achieve in high-dimensional data. It uses new variables to represent the original variables. These new variables do not lose useful information in the original variables as much as possible (Rezazad Bari et al., 2021). The new variables are called Principal Components (PC).

#### Pre-processing Methods

Experimentally acquired THz spectra often contain some interferences from factors unrelated to the nature of the sample. These interferences can cause baseline drift of the spectrum and generate random noise, etc. At the same time, the absorption peaks often appear to overlap. Therefore, it is essential for the spectral data to be subjected to pre-processing methods. The pre-processing method can amplify the original hidden signal differences in the spectral data. Meanwhile, spectral pre-processing techniques can achieve the purpose of improving the resolution of THz spectral data, making the identification more accurate and reliable (Ndlovu et al., 2021; Tafintseva et al., 2021). In this paper, seven pre-processing methods were used, including: mean-centering, auto scaling, standard normal variate (SNV), minimum and maximum values to [0 1], multiplicative scatter correction (MSC), first derivative, and second derivative (Lu, 2006; Chu, 2011).

#### Discriminant Partial Least Squares

Discriminant Partial Least Squares (DPLS) is a discriminant analysis method based on PLSR (Lei et al., 2021). It is a widely used method for supervised pattern discrimination. This method considers the experimental sample characteristics data as the independent variables *X* (whose rows are the sample ordinal numbers and columns are the characteristic variable ordinal numbers). The category information of the experimental samples is considered as the dependent variable *Y*. *Y* is a matrix composed of 0, 1. The rows correspond to the sample numbers. The columns correspond to the category serial numbers. When a sample belongs to a category, the element value of the corresponding column in *Y* is 1. Otherwise, it is 0. In order to decide the problem of attribution of a substance in a mixture, the category matrix must be able to describe a specific kind of sample (Xue et al., 2021). DPLS is commonly applied in cases where the number of variables is high and there are multiple commonalities.

#### Particles Swarm Optimization-Support Vector Machine

SVM is a very widely used pattern recognition model proposed based on statistical theory (Wang et al., 2021b). In this paper, radial basis function was used as the kernel function of SVM. The classification hyperplane established by SVM can guarantee the classification accuracy (Wang et al., 2021a). For the optimization problem of the parameters of the SVM (parameter *c* and *g*), this paper used the particles swarm optimization (PSO) algorithm (Huang et al., 2021) and the grey wolf optimizer (GWO) algorithm (Deng et al., 2021). PSO is an optimization algorithm for group intelligence. It is derived from the study of predatory behavior of birds. The basic idea of the PSO algorithm is to find the optimal solution through collaboration and information sharing among individuals in a population (Zhou et al., 2021b). Each particle in this algorithm represents a potential solution to the problem. The velocity of the particle is dynamically adjusted with the movement experience of itself and other particles, thus achieving individual optimality search in the solvable space.

#### Grey Wolf Optimizer-Support Vector Machine

GWO is a meta-heuristic optimization algorithm. It has a more reasonable global optimal solution search mechanism, greater operational stability, and faster convergence than other optimization algorithms (Liu et al., 2021). The GWO algorithm is proposed based on imitating the hunting process of a wolf pack. It is mainly divided into three steps, which are encirclement, hunting, and attack. The highest rank in this algorithm is the head wolf, with two of them, marked as α. The head wolf is responsible for making decisions and leading the pack during the hunting (finding the optimal parameters) process. The remaining wolves are, respectively, labeled β, δ, and ω from top to bottom according to rank. The behavior of the next rank follows the leadership of the previous rank. Firstly, the wolves encircle the target during the hunting process. After encircling the prey, the wolves perform hunting behavior. The process is usually led by α, β, and δ. Other search units (ω) should update their respective positions according to the current position of the best search unit. Finally, the wolves attack the prey and accomplish the goal of capturing the prey (Zhou et al., 2021a).

DPLS, PSO-SVM, and GWO-SVM soybean variety identification models were established and predicted done in MATLAB R2018a. The computer operating system was Windows 10.0. The CPU was i7 8750 H. The memory is 16 g 2,666. In this paper, the correct identification rate was calculated in the same way as the accuracy.

## Results and Discussion

### Terahertz Frequency-Domain Spectra

**FIGURE 2:**
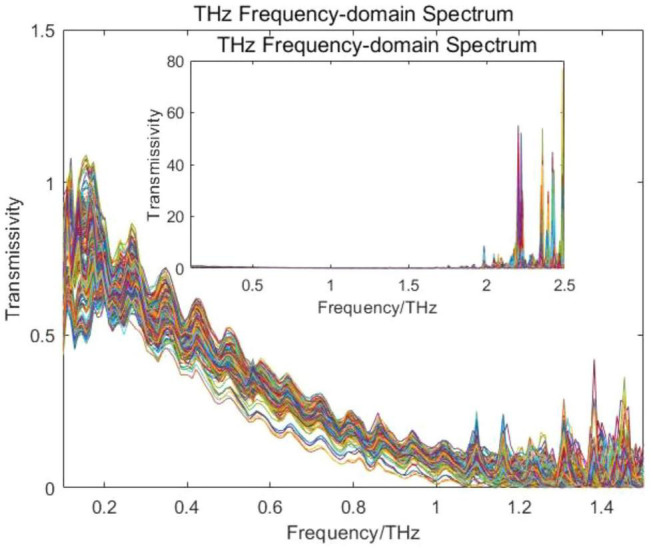
THz frequency-domain spectra of different variety experimental samples.

Figure 2 shows the THz frequency-domain spectral images of the experimental samples in the interval of 0.1–1.5 THz and 0.1–2.5 THz. The effective range of THz spectra measured by the THz spectroscopy equipment used in this experiment was from 0.1 to 2.5 THz. When the THz spectral frequency was between 1.5 and 2.5 THz, the signal-to-noise ratio of the spectrum was too low to be selected. Hence, the 0.1–1.5 THz interval was selected as the modeling spectral interval for the soybean variety identification model in this experiment. It could not be seen from Figure 2 that there were significant differences in the THz spectra of the different variety experiment samples. Therefore, the THz spectra of the experimental samples should be analyzed and identified in combination with chemometrics.

### Principal Component Analysis

**FIGURE 3:**
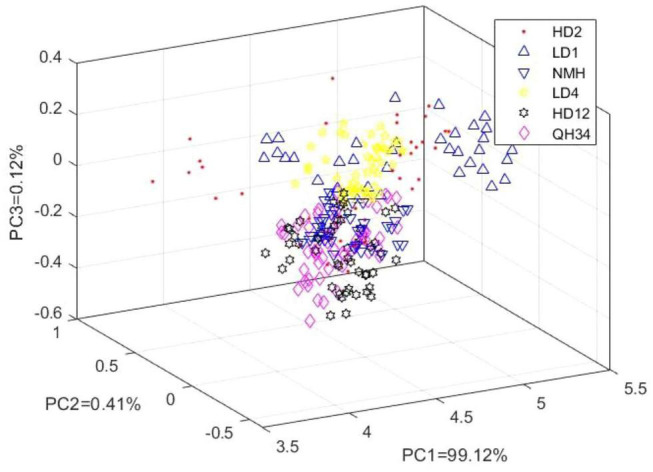
The PC score plot.

The THz frequency-domain spectra of the samples were subjected to PCA. The cumulative variance contribution of the first 3 PCs was 99.65%. Therefore, the information of the distribution characteristics of the samples could be basically characterized by the projected distribution of the first 3 PCs in space. The PC score plot is shown in Figure 3. From Figure 3, it could be found that the experimental samples of HD_2_ and LD_1_ were distributed more scattered in the three-dimensional space. However, the remaining four varieties of experimental samples showed obvious overlap in the distribution in three-dimensional space. In the overlapping part, it was very difficult to distinguish and identify the experimental sample varieties using the naked eye. Therefore, good results could not be obtained by using PCA alone to identify soybean varieties. Thus, THz spectroscopy required the use of identification methods with supervised modes for soybean variety identification.

### Variety Identification Model Establishment and Validation

#### Establishment and Validation of the Discriminant Partial Least Squares Soybean Variety Identification Model

The transmissibility and frequency of the spectral points in the selected frequency interval were used as the input matrix. The DPLS was used to establish the soybean variety identification model. The 270 experimental samples were divided according to the ratio of calibration set to test set of 3:1. Therefore, 67 samples were randomly selected as the test set. The remaining 203 samples were used as the calibration set. The 67 test set samples contained 10 HD_2_, 10 LD_1_, 7 NMH, 13 LD_4_, 16 HD_12_, and 11 QH_34_. The 203 calibration set samples contained 26 HD_2_, 26 LD_1_, 29 NMH, 41 LD_4_, 38 HD_12_, and 43 QH_34_. Firstly, the THz spectra were separately subjected to seven pre-processing methods. Secondly, the DPLS soybean variety identification model was established by the calibration set. Finally, the effects of the variety identification model were validated using the test set. The validation results are shown in Table 1.

**TABLE 1:** DPLS soybean variety identification model validation results.

Spectral pre-processing methods	The best number of PC	Correct identification rate%							Overall precision%	Overall F1 score%	Identification time used s
		HD_2_	LD_1_	NMH	LD_4_	HD_12_	QH_34_	Overall			
(a).None	9	40	60	85.71	100	68.75	100	76.12	79.18	75.64	0.90
(b).Mean-centering	10	40	80	85.71	100	68.75	100	79.10	84.28	78.64	0.51
(c).Auto scaling	10	50	60	85.71	100	75	100	79.10	80.96	78.66	0.35
(d).SNV	9	20	70	85.71	100	68.75	100	74.63	81.66	72.88	0.47
(e).Minimum and maximum values to [0 1]	10	50	50	85.71	100	81.25	100	79.10	79.35	78.40	0.47
(f).MSC	9	20	70	85.71	100	68.75	100	74.63	81.66	72.88	0.79
(g).First derivative	10	50	70	85.71	100	56.25	100	76.12	76.06	82.13	0.65
**(h).Second derivative**	**9**	**50**	**70**	**100**	**100**	**68.75**	**100**	**80.60**	**85.79**	**80.42**	**0.62**

*The row with the highest value of overall correct identification rate% and precision% is highlighted in bold.*

From Table 1, it could be found that the results of the DPLS soybean variety identification model were not very satisfactory. The overall correct identification rate was in the range of 74–81%. The validation results of the DPLS soybean variety identification model showed relatively obvious changes after the THz frequency-domain spectra were subjected to different pre-processing methods. In terms of the overall correct identification rate, the overall correct identification rate of the DPLS soybean variety identification model could be improved to 80.60% after the second derivative pre-processing of the THz spectra. This was a 4.48% improvement compared to the DPLS soybean variety identification model without the pre-processing method, and the identification time used was relatively shorter. At the same time, the overall precision of this identification model achieved the highest value. This might be because the second derivative pre-processing method could effectively eliminate the interference of baseline and other backgrounds. Judging from the identification of different variety soybeans, the DPLS soybean variety identification model was very effective in identifying LD_4_ and QH_34_. The DPLS soybean variety identification model was significantly improved for NMH by the second derivative pre-processing. The DPLS soybean variety identification model was not well for the identification of three soybean varieties (HD2, LD1, and HD12). Compared to other varieties, the identification results of these three varieties of soybean needed to be further improved.

#### Establishment and Validation of the Particles Swarm Optimization-Support Vector Machine and Grey Wolf Optimizer-Support Vector Machine Soybean Variety Identification Models

The experimental samples were divided into the calibration and test set according to the same method as before. The calibration set was formed by 203 experimental samples. The PSO-SVM and GWO-SVM soybean variety discrimination models were established using the calibration set. The test set was composed of 63 experimental samples. Validation of the soybean variety discrimination models was performed using the test set. The parameters *c* and *g* were, respectively, optimized by the PSO algorithm and GWO algorithm. The validation results of the two soybean variety identification models are shown in Tables 2, 3.

**TABLE 2:** PSO-SVM soybean variety identification validation results.

Spectral pre-processing methods	Correct identification rate%							Overall precision%	Overall F1 score%	Identification time used s
	HD_2_	LD_1_	NMH	LD_4_	HD_12_	QH_34_	Overall			
None	100	90	100	92.31	75	100	91.04	93.35	91.29	196.04
(b). Mean-centering	100	90	100	92.31	75	100	91.04	93.35	91.29	230.47
**(c). Auto scaling**	**100**	**90**	**100**	**100**	**75**	**100**	**92.54**	**94.84**	**92.77**	**331.03**
(d). SNV	90	90	85.71	100	75	100	89.55	91.11	89.81	239.65
(e). Minimum and maximum values to [0 1]	100	80	100	100	75	100	91.04	93.71	91.25	278.69
(f). MSC	90	90	85.71	100	75	100	89.55	91.11	89.81	152.84
(g). First derivative	90	80	100	92.31	81.25	100	89.55	90.90	89.72	211.92
(h). Second derivative	100	80	71.43	100	87.5	100	91.04	91.44	90.99	248.08

*The row with the highest value of overall correct identification rate% and precision% is highlighted in bold.*

**TABLE 3:** GWO-SVM soybean variety identification validation results.

Spectral pre-processing methods	Correct identification rate%							Overall precision%	Overall F1 score%	Identification time used s
	HD_2_	LD_1_	NMH	LD_4_	HD_12_	QH_34_	Overall			
(a). None	100	90	100	84.62	75	100	89.55	92.13	89.81	162.33
(b). Mean-centering	100	90	100	84.62	75	100	89.55	92.13	89.81	147.19
(c). Auto scaling	100	90	100	100	75	100	92.54	94.84	92.77	330.74
(d). SNV	90	100	85.71	92.31	75	100	89.55	91.50	89.89	218.83
(e). Minimum and maximum values to [0 1]	100	80	100	100	75	100	91.04	93.71	91.25	322.81
(f). MSC	90	100	85.71	92.31	75	100	89.55	91.50	89.89	182.52
(g). First derivative	100	100	100	92.31	81.25	100	94.03	95.51	94.20	160.37
**(h). Second derivative**	**100**	**100**	**85.71**	**100**	**93.75**	**100**	**97.01**	**97.01**	**97.01**	**181.66**

*The row with the highest value of overall correct identification rate% and precision% is highlighted in bold.*

From Tables 2, 3, it could be found that after the first derivative and the second derivative pre-processing methods, the overall correct identification rate of the GWO-SVM soybean variety identification model was very significantly improved compared to the PSO-SVM variety identification model. The identification time used was also significantly reduced. Meanwhile, the overall precision of the GWO-SVM variety identification model achieved the highest value. This might be because the parameter optimization of the SVM by GWO imitated the wolf hunting process so that it could obtain a more reasonable global optimal solution search capability. Therefore, the GWO algorithm showed superior soybean variety identification performance compared to the PSO algorithm in finding the optimal parameters of the SVM. This was of great practical importance for soybean variety identification. The overall correct identification rate of the GWO-SVM soybean variety identification model (*c* = 7.77 × 10^9^, *g* = 7.95 × 10^–4^) was improved to 97.01% after the second derivative pre-processing method for the THz frequency-domain spectra. The identification time used of the variety identification model was 181.66 s. This indicated that THz frequency-domain spectroscopy combined with chemometrics could quickly and accurately identify soybean varieties. After THz frequency-domain spectra were preprocessed with the second derivative, the GWO-SVM variety identification model improved the overall correct identification rate by 7.46% compared to the GWO-SVM identification model without the pre-processing method. This further indicated that the second derivative pre-processing method played an important role in eliminating background interference, resolving overlapping peaks, etc. for the THz spectra. Thus, the second derivative pre-processing method best improved the overall correct identification rate of the GWO-SVM identification model. After the second derivative pre-processing, the GWO-SVM identification model could reach 100% for four varieties (HD_2_, LD_1_, LD_4_, and QH_34_) of soybeans. The correct identification rate of the other two varieties of soybeans also reached more than 85%. By observing the validation results of the two variety identification models combined with the seven pre-processing methods, it was easy to see that the THz spectra combined with different pre-processing methods had a great impact on the correct identification rate of the identification model and the time used for identification. Therefore, it was crucial to choose the appropriate pre-processing method for different identification models.

Comparing Tables 1–3, it was found that the overall correct identification rate of the GWO-SVM soybean variety identification model was better than that of the DPLS and PSO-SVM variety identification models after the first derivative and second derivative preprocessing methods. However, the DPLS soybean variety identification model took significantly shorter time to identify than the other two variety identification models. When comparing the validation results of the DPLS, PSO-SVM, and GWO-SVM identification models, it was found that the THz spectra combined with the second derivative pre-processing method resulted in the best identification results and relatively short identification time for the GWO-SVM variety identification model. The overall correct identification rate was 97.01% (85.71% for NMH, 93.75% for HD_12_, and 100% for others), and the identification time used was 181.66 s. However, there were some limitations of this variety identification model. The identification model needed to be continuously optimized for different THz spectral data. For soybean varieties for which THz spectral data characteristics had been not collected, the identification model identified relatively poor results.

## Conclusion

The experimental results showed that it was feasible to identify soybean varieties by THz frequency-domain spectroscopy combined with chemometrics. The GWO-SVM soybean variety identification model achieved the best results and relatively short identification time used after the second derivative pre-processing method for THz spectra. The overall correct identification rate was 97.01% and the identification time used was 181.66 s. This indicated that this method was an accurate means of identifying soybean varieties. The identification time used was relatively short, and the identification speed was relatively fast. In addition, the DPLS and PSO-SVM variety identification models combined with suitable pre-processing methods could also be used for soybean variety identification. The novelty of the paper was that the feasibility of THz frequency-domain spectroscopy combined with chemometrics for soybean variety identification was analyzed and investigated. At the same time, a soybean variety identification model based on the GWO-SVM was proposed. The study has some reference value for the rapid and accurate identification of agricultural products and food varieties based on THz spectroscopy.

## Data Availability Statement

The original contributions presented in the study are included in the article/supplementary material, further inquiries can be directed to the corresponding author/s.

## Author Contributions

XW: conceptualization, methodology, software, validation, formal analysis, investigation, writing—review and editing, writing—original draft, and visualization. DdK: investigation, resources, data curation, writing—review and editing, and supervision. SpZ: definition, validation, resources, data curation, writing—review and editing, project, administration, and funding. SL: formal analysis and investigation. SlZ: resources, data curation, and administration. WjW: investigation, resources, and data curation. All authors contributed to the article and approved the submitted version.

## Conflict of Interest

The authors declare that the research was conducted in the absence of any commercial or financial relationships that could be construed as a potential conflict of interest.

## Publisher’s Note

All claims expressed in this article are solely those of the authors and do not necessarily represent those of their affiliated organizations, or those of the publisher, the editors and the reviewers. Any product that may be evaluated in this article, or claim that may be made by its manufacturer, is not guaranteed or endorsed by the publisher.
